# Supplementary material for: GluN2A and GluN2B NMDA receptors use distinct allosteric routes
Source: Nat Commun. 2021 Aug 5;12:4709. doi: 10.1038/s41467-021-25058-9 (PMC8342458; doi:10.1038/s41467-021-25058-9)
Supplement: Supplementary file 1 — Supplementary Information [file 41467_2021_25058_MOESM1_ESM.pdf]

GluN2A and GluN2B NMDA receptors use distinct allosteric routes

Meilin Tian, David Stroebel, Laura Piot, Mélissa David, Shixin Ye and Pierre Paoletti

Correspondence to:
Dr. Shixin Ye, shixin.ye-lehmann@inserm.fr; Dr. Pierre Paoletti, pierre.paoletti@ens.psl.eu

Supplementary Table 1

Supplementary Figures 1-9

Legend of Movie

Supplementary References

Supplementary Table 1: Dataset of GluN1/GluN2 structures used for the structural analysis presented in Fig. 6. Reso stands for resolution. * indicates one of the two NTD zinc sites mutated.

PDB code	Receptor state	Technic/ reso (Å)	Conditions	Corresponding construct	Reference
4PE5	**2B** Inhibited	X-ray / 4.0	Glu+Gly, pH 8.8, Ifenprodil	No CTD, multiple mutations	Karakas et al. 2014[1]
4TLL	**2B** Inhibited	X-ray / 3.6	ACPC+ACBD, pH 7.5, Ro25-6981, MK-801	No CTD, multiple mutations	Lee et al. 2014[2]
4TLM	**2B** Inhibited	X-ray / 3.8	ACPC+ACBD, pH 7.5, Ro25-6981, MK-801	No CTD, multiple mutations	Lee et al. 2014[2]
5IOV	**2B** Inhibited	EM / 7.5	Glu+Gly, pH 6.5, Ro25-6981	No CTD, multiple mutations	Zhu et al. 2016[3]
5FXH	**2B** Non-Act	EM / 5.0	Glu+Gly, pH 7.3	No CTD, multiple mutations	Tajima et al. 2016[4]
5FXI	**2B** Non-Act	EM / 6.4	Glu+Gly, pH 7.3	No CTD, multiple mutations	Tajima et al. 2016[4]
5FXJ	**2B** Non-Act	EM / 6.3	Glu+Gly, pH 7.3	No CTD, multiple mutations	Tajima et al. 2016[4]
5FXK	**2B** Non-Act	EM / 6.4	Glu+Gly, pH 7.3	No CTD, multiple mutations	Tajima et al. 2016[4]
6CNA	**2B** Non-Act	EM / 4.6	Glu+Gly, pH 7.3	No CTD + Exon 5	Regan et al. 2018[5]
6WHR	**2B** Non-Act	EM / 4.0	Glu+Gly, pH 7.3	No CTD	Chou et al. 2020[6]
6WHS	**2B** Non-Act	EM / 4.0	Glu+Gly, pH 7.3	No CTD	Chou et al. 2020[6]
5IOU	**2B** Non-Act	EM / 7.0	Glu+Gly, pH 6.5	No CTD, multiple mutations	Zhu et al. 2016[3]
5FXG	**2B** Pre-active	EM / 6.8	Glu+Gly, pH 7.3	No CTD, multiple mutations (map with no TMD)	Tajima et al. 2016[4]
6WHT	**2B** Pre-active	EM / 4.4	Glu+Gly, pH 7.3	No CTD	Chou et al. 2020[6]
6WI1	**2B** Pre-active	EM / 3.6	Glu+Gly, pH 7.3	No CTD mutant locked in unrolled state	Chou et al. 2020[6]
6MMA	**2A** Inhibited (Ext)	EM / 6.3	Glu+Gly, pH 6.1, 1 μM $ZnCl_2$	No CTD	Jalali-Yazdi et al. 2018[7]
6MMX	**2A** Inhibited (Ext)	EM / 7.0	Glu+Gly, pH 7.4, 1 μM $ZnCl_2$	No CTD*	Jalali-Yazdi et al. 2018[7]
6MMM	**2A** Inhibited (Ext)	EM / 6.8	Glu+Gly, pH 7.4, 1 μM $ZnCl_2$	No CTD	Jalali-Yazdi et al. 2018[7]
6MMH	**2A** Inhibited (Ext)	EM / 8.2	Glu+Gly, pH 7.4, 1 mM $ZnCl_2$	No CTD	Jalali-Yazdi et al. 2018[7]
6MM9	**2A** Inhibited (1K)	EM / 6.0	Glu+Gly, pH 6.1, 1 μM $ZnCl_2$	No CTD	Jalali-Yazdi et al. 2018[7]
6MMK	**2A** Inhibited (1K)	EM / 6.1	Glu+Gly, pH 7.4, 1 μM $ZnCl_2$	No CTD	Jalali-Yazdi et al. 2018[7]
6MMT	**2A** Inhibited (1K)	EM / 7.5	Glu+Gly, pH 7.4, 1 μM $ZnCl_2$	No CTD*	Jalali-Yazdi et al. 2018[7]
6IRF	**2A** Inhibited (1K)	EM / 5.1	Glu+Gly, pH 6.3, EDTA	No CTD, multiple mutations	Zhang et al. 2018[8]
6IRA	**2A** Pre-act 2KA	EM / 4.5	Glu+Gly, pH 7.8, EDTA	No CTD, multiple mutations	Zhang et al. 2018[8]
6MMU	**2A** Pre-act 2KA	EM / 5.3	Glu+Gly, pH 7.4, 1 μM $ZnCl_2$	No CTD*	Jalali-Yazdi et al. 2018[7]
6MMV	**2A** Pre-act 2KA	EM / 4.7	Glu+Gly, pH 7.4, 1 μM $ZnCl_2$	No CTD* (map with no TMD)	Jalali-Yazdi et al. 2018[7]
6MML	**2A** Pre-act 2KA	EM / 7.1	Glu+Gly, pH 7.4, 1 μM $ZnCl_2$	No CTD	Jalali-Yazdi et al. 2018[7]
6MMS	**2A** Pre-act 2KS	EM / 5.4	Glu+Gly, pH 7.4, 1 mM EDTA	No CTD*	Jalali-Yazdi et al. 2018[7]
6MMR	**2A** Pre-act 2KS	EM / 5.1	Glu+Gly, pH 7.4, 1 mM $ZnCl_2$, 3 mM EDTA	No CTD	Jalali-Yazdi et al. 2018[7]
6MMG	**2A** Pre-act 2KS	EM / 6.2	Glu+Gly, pH 7.4, 1 mM EDTA	No CTD	Jalali-Yazdi et al. 2018[7]

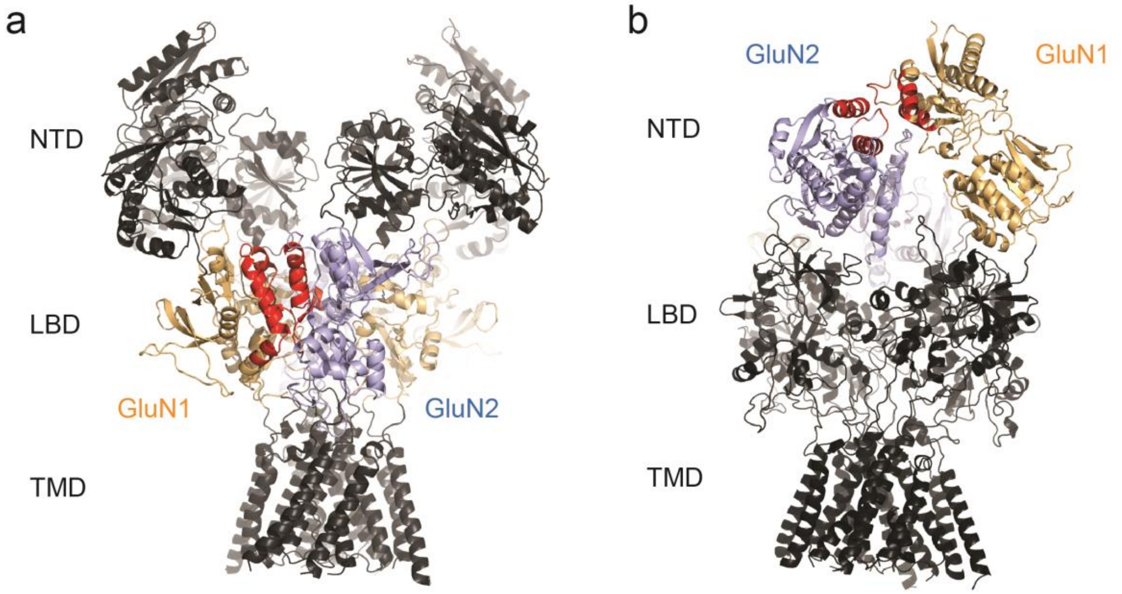

c

	GluN2A *vs* GluN2B % identity/homology*	GluN2A *vs* GluN2C % identity/homology*	GluN2A *vs* GluN3A % identity/homology*	GluN2A *vs* GluN1 % identity/homology*
Entire sequence	54 / 65	45 / 53	22 / 34	23 / 37
LBD only	82 / 89	75 / 81	37 / 50	36 / 49
LBD Intra-dimer interface only (67 AA) – *panel A*	**94 / 100**	93 / 97	51 / 57	43 / 57
NTD only	53 / 69	36 / 48	20 / 32	18 / 31
NTD Intra-dimer interface only (37 AA) – *panel B*	**76 / 86**	48 / 65	24 / 35	16 / 24

*Blosum62

Supplementary Figure 1: High sequence conservation between GluN2A and GluN2B receptors at key domain-domain interfaces.

(a) Location of the LBD intra-dimer interface (red) in the GluN1/GluN2 tetramer structure.

(b) Location of the NTD intra-dimer interface (red) in the GluN1/GluN2 tetramer structure.

(c) Amino acid (AA) sequence identity and homology (Blosum62) between GluN2A and other NMDAR subunits for different region of the receptor. In red are indicated the identity and homology values between GluN2A and GluN2B subunits at the interfaces highlighted in panels a and b.

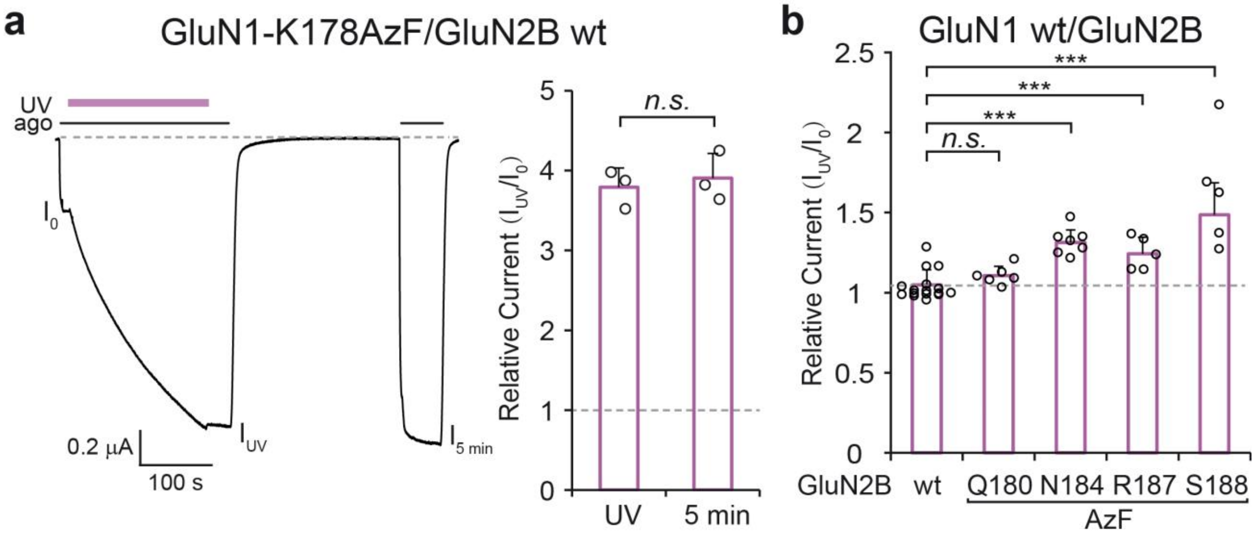

Supplementary Figure 2: Stability of the photopotentiation and UV potentiation of GluN2B AzF mutant receptors.

(a) The UV-induced photopotentiation of GluN1-K178AzF/GluN2B receptors is stable over prolonged periods of times (minutes). Left panel: representative current traces measured from an oocyte expressing GluN1-K178AzF/GluN2B receptors during UV illumination (365 nm) and 5 min after the illumination. Right panel: change in current amplitude immediately after UV illumination (left bar) and after five additional minutes of recording in the absence of UV (right bar). Values are: 3.79 ± 0.24 (n=3) and 3.90 ± 0.31 (n=3), respectively. Data represent mean ± SD. n=number of biologically independent cells. *n.s*. non-significant (P=0.64) (two-sided Student's t-test).

(b) Change in current amplitude upon UV illumination (I_{uv}/I_o) of wild-type (wt) GluN1/GluN2B receptors and various GluN1/GluN2B-AzF mutant receptors. Values are: 1.04 ± 0.09 (n=16) for wt, 1.11 ± 0.06 (n=6) for GluN2B-Q180AzF, 1.32 ± 0.08 (n=7) for GluN2B-N184AzF, 1.24 ± 0.10 (n=5) for GluN2B-R187AzF, 1.62 ± 0.35 (n=5) for GluN2B-S188AzF. Data represent mean ± SD. n=number of biologically independent cells. ***P<0.001, *n.s*. non-significant (P=0.10 for GluN2B-Q180AzF) (one-way ANOVA).

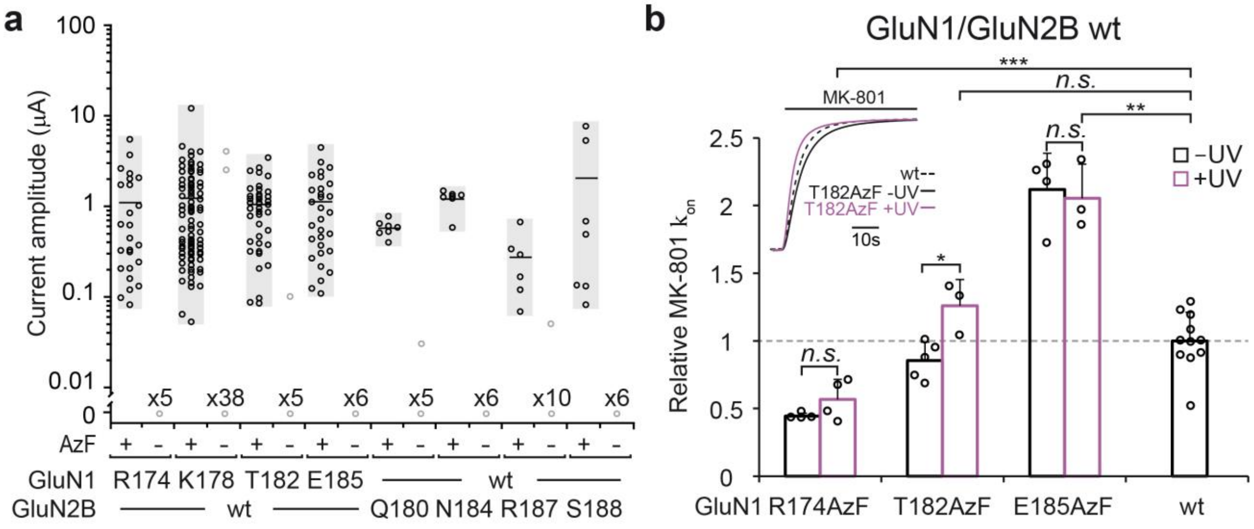

Supplementary Figure 3: Efficient AzF incorporation and assessment of channel activity of GluN1-AzF/GluN2B mutant receptors.

(a) Current amplitudes measured from oocytes expressing various AzF mutant receptors and incubated in the presence '+' or absence '-' of 1 mM AzF. Current of at least 5 oocytes in each condition were tested and plotted. For the group '+ AzF', only oocytes with measurable currents (>10 nA) were plotted. wt, wild-type. Dot plot: grey squares represent the range of current amplitudes and the lines the mean values. Values of n (number of biologically independent cells) are indicated.

(b) Assessment of receptor channel activity using MK-801 inhibition kinetics. MK-801 k_{on} values were normalized to the mean value obtained with wild-type (wt) GluN1/GluN2B receptors. Relative values are, from left to right: 0.44 ± 0.02 (n=4) without UV and 0.57 ± 0.15 (n=4) with UV for GluN1-R174AzF; 0.85 ± 0.13, (n=5) without UV and 1.26 ± 0.19 (n=3) with UV for GluN1-T182AzF; 2.12 ± 0.27 (n=4) without UV and 2.05 ± 0.25 (n=3) with UV for GluN1-E185AzF; 1.00 ± 0.21 (n=11) for wt (no UV). Inset: representative scaled current traces from oocytes expressing wild-type (dashed) or GluN1-T182AzF before (black) and after (violet) UV illumination in response to 50 nM MK-801. Data represent mean ± SD. n=number of biologically independent cells. *P=0.012, **P=0.005, ***P<0.001, *n.s.* non-significant (P=0.15 for GluN1-R174AzF and 0.76 for GluN1-E185AzF, before and after UV; P=0.08 for GluN1-T182AzF after UV compared with wt) (one-way ANOVA).

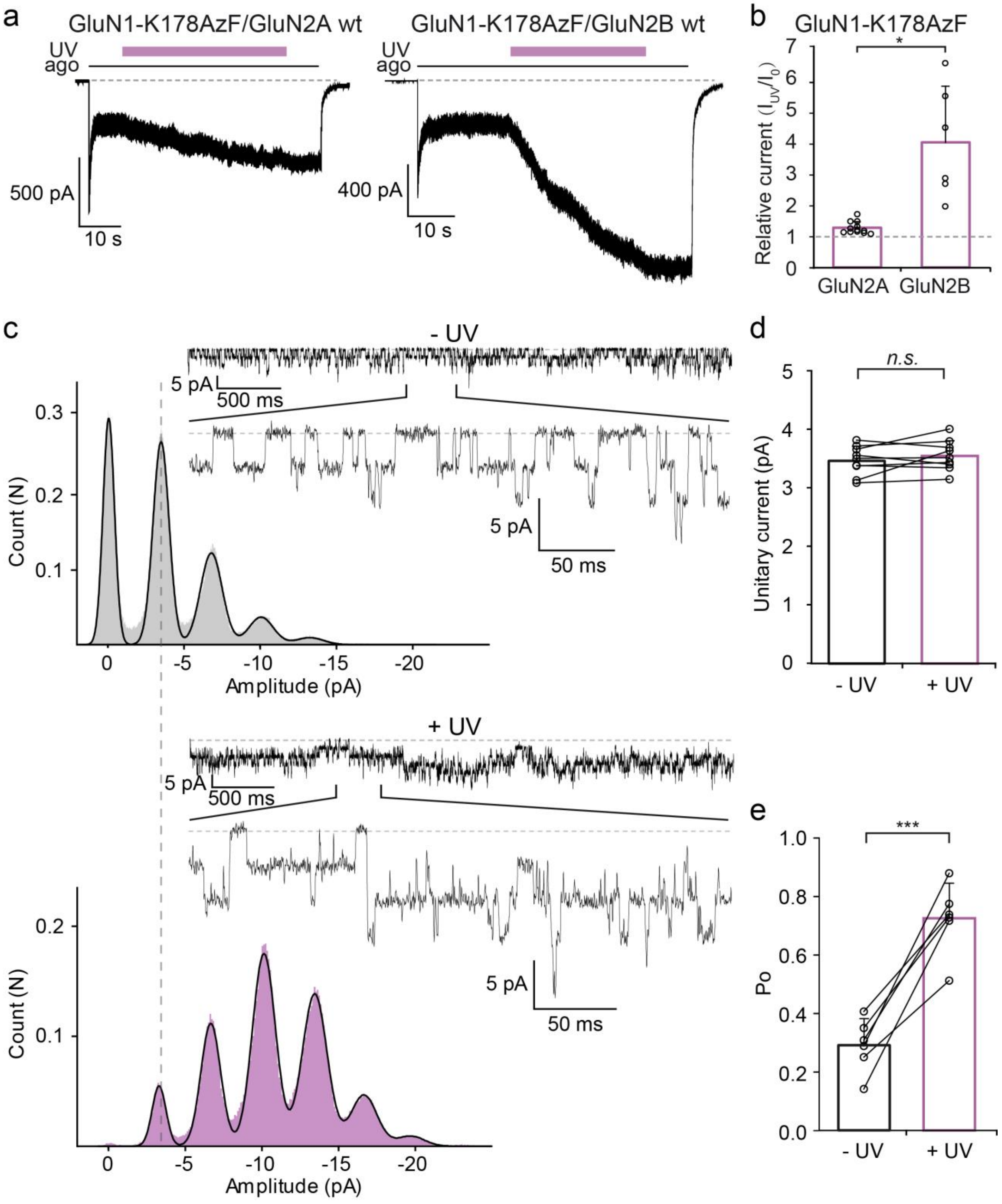

Supplementary Figure 4: Photo-potentiation of GluN1-K178AzF receptors expressed in HEK cells at the macroscopic and single-channel levels.

(a) Representative current traces measured from HEK cells expressing GluN1-K178AzF/GluN2A (left) and GluN1-K178AzF/GluN2B (right) receptors during UV illumination (365 nm) in the presence of agonists (ago, 100 µM glutamate + 100 µM glycine).

(b) Change in current amplitude upon UV illumination (I_{uv}/I_o) of GluN1-K178AzF/GluN2A and GluN1-K178AzF/GluN2B receptors expressed in HEK cells. Values are: 1.30 ± 0.19 (n=12) for GluN1-K178AzF/GluN2A, 4.06 ± 1.82 (n=6) for GluN1-K178AzF/GluN2B. Data represent mean ± SD. n=number of biologically independent cells. *P=0.013 (two-sided Student's t-test).

(c) Representative single-channel recordings from an outside-out patch expressing GluN1-K178AzF/GluN2B receptors before (top) and after (bottom) UV illumination. Time-expanded views are also displayed. All-points amplitude histograms from at least 17 s of recordings in both conditions (same patch) are shown. Data (grey and violet filling before and after UV, respectively) were fitted with multiple Gaussian components (dark lines). Data were filtered at 1 kHz for display.

(d) UV illumination does not affect unitary conductance of GluN1-K178AzF/GluN2B receptors. Values of unitary currents are: 3.46 ± 0.25 pA (n= 9) before UV and 3.55 ± 0.26 pA (n=9) after UV (P=0.28; two-sided paired t-test). Data represent mean ± SD. n=number of biologically independent cells.

(e) UV illumination locks GluN1-K178AzF/GluN2B receptors in a high open probability (Po) mode. Increase of channel Po before and after UV treatment. Values are calculated from whole-cell noise analysis combined with measurements of unitary currents as illustrated in panel d (see Methods). Po values are: 0.29 ± 0.09 (n= 6) before UV and 0.73 ± 0.12 (n=6) after UV (*** P<0.001; two-sided paired t-test). Data represent mean ± SD. n=number of biologically independent cells.

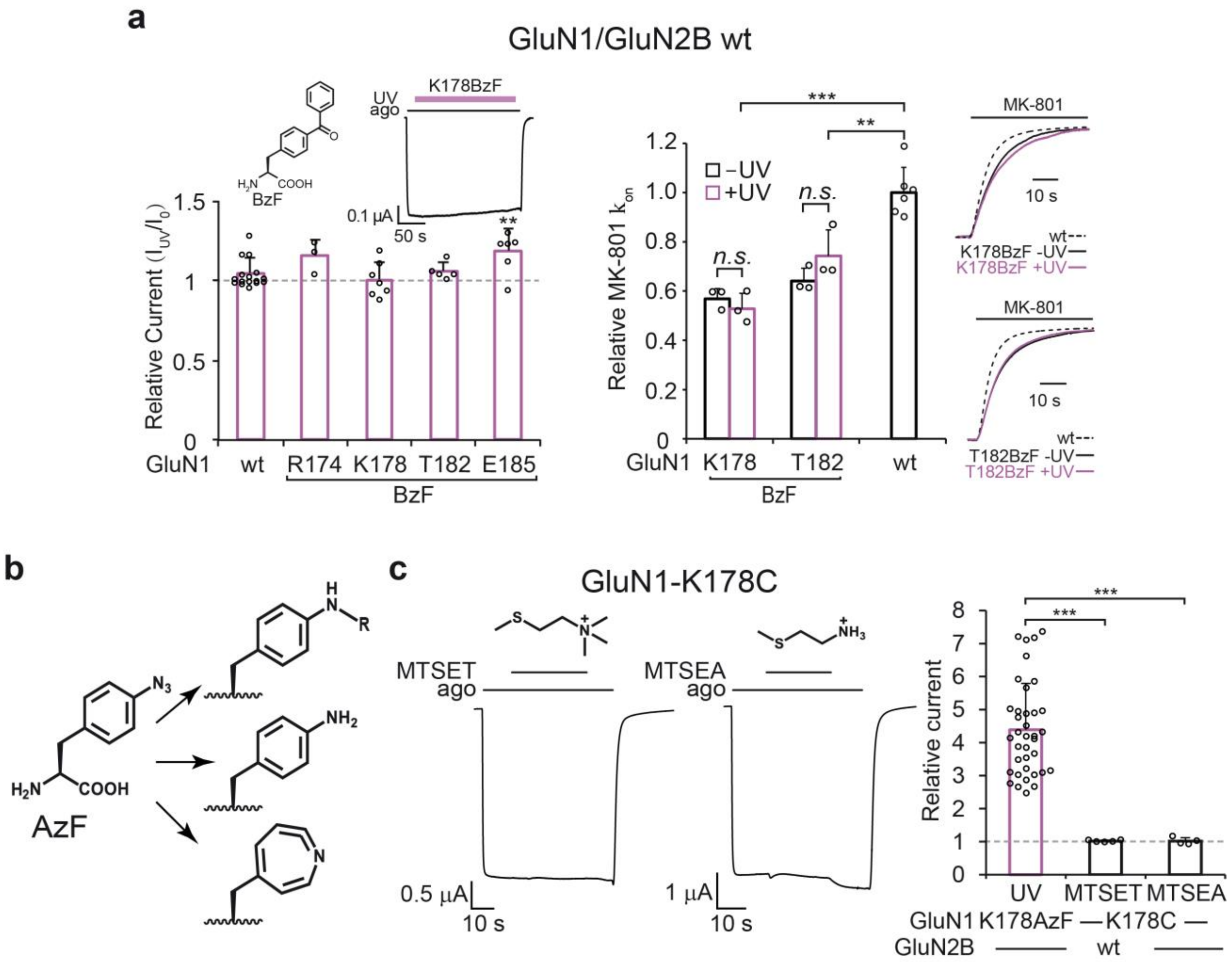

Supplementary Figure 5: Photo-responsiveness of GluN1-BzF/GluN2B mutant receptors and MTS modification of GluN1-K178C/GluN2B receptors.

(a) Left panel: change in current amplitude upon UV illumination (I_{uv}/I_o) of wild-type (wt) GluN1/GluN2B receptors and various GluN1-BzF/GluN2B mutant receptors. Values are: 1.04 ± 0.09 (n=16) for wt, 1.15 ± 0.10 (n=3) for GluN1-R174BzF, 1.01 ± 0.11 (n=7) for GluN1-K178BzF, 1.06 ± 0.05 (n=5) for GluN1-T182BzF and 1.19 ± 0.14 (n=6) for GluN1-E185BzF. Inset: representative current trace measured from oocytes expressing GluN1-K178BzF mutant receptor during UV illumination. Error bars represent the standard deviation. The mutants are non-significant comparing with wt GluN1/GluN2B receptors: P=0.051 for GluN1-R174BzF, 0.47 for GluN1-K178BzF, 0.61 for GluN1-T182BzF, except **P=0.009 for GluN1-E185BzF (one-way ANOVA). Right panel: Assessment of receptor channel activity using MK-801 inhibition kinetics. MK-801 k_{on} values were normalized to the mean value obtained with wild-type (wt) GluN1/GluN2B receptors. Relative values are, from left to right: 0.57 ± 0.04 (n=3) without UV and 0.53 ± 0.06 (n=3) with UV for GluN1-K178BzF; 0.64 ± 0.05, (n=3) without UV and 0.74 ± 0.11 (n=3) with UV for GluN1-T182BzF; 1.00 ± 0.10 (n=6) for wt (no UV). Inset: representative

scaled current traces from oocytes expressing wild-type (dashed) or GluN1-K178BzF (top) and GluN1-T182BzF (bottom) before (black) and after (violet) UV illumination in response to 50 nM MK-801. Data represent mean ± SD. n=number of biologically independent cells. **P=0.009, ***P<0.001, *n.s.* non-significant (P=0.41 for GluN1-K178BzF and 0.21 for GluN1-T182BzF, before and after UV) (one-way ANOVA).

(b) Multiple photochemical pathways of p-azido-L-phenylalanine (AzF). Besides the photo-crosslinking reaction (right top), upon UV illumination the nitrene can undergo reduction to an amine (right middle). It can also undergo ring expansion to form a 7-membered cyclic ketenimine (right bottom). Adapted from ref[9].

(c) MTS effects on GluN1-K178C/GluN2B receptors. Left panel: representative current trace measured from oocytes expressing GluN1-K178C/GluN2B receptors during application MTSET or MTSEA. Right panel: change in current amplitude following MTSET (200µM) and MTSEA (200 µM) treatment. Values are: 1.02 ± 0.03 (n=5) and 1.01 ± 0.11 (n=4), respectively, to be compared with the extent of photopotentiation observed at GluN1-K178AzF/GluN2B receptors (4.39 ± 1.40 [n=37]) upon UV illumination. Data represent mean ± SD. n=number of biologically independent cells. ***P<0.001 (one-way ANOVA).

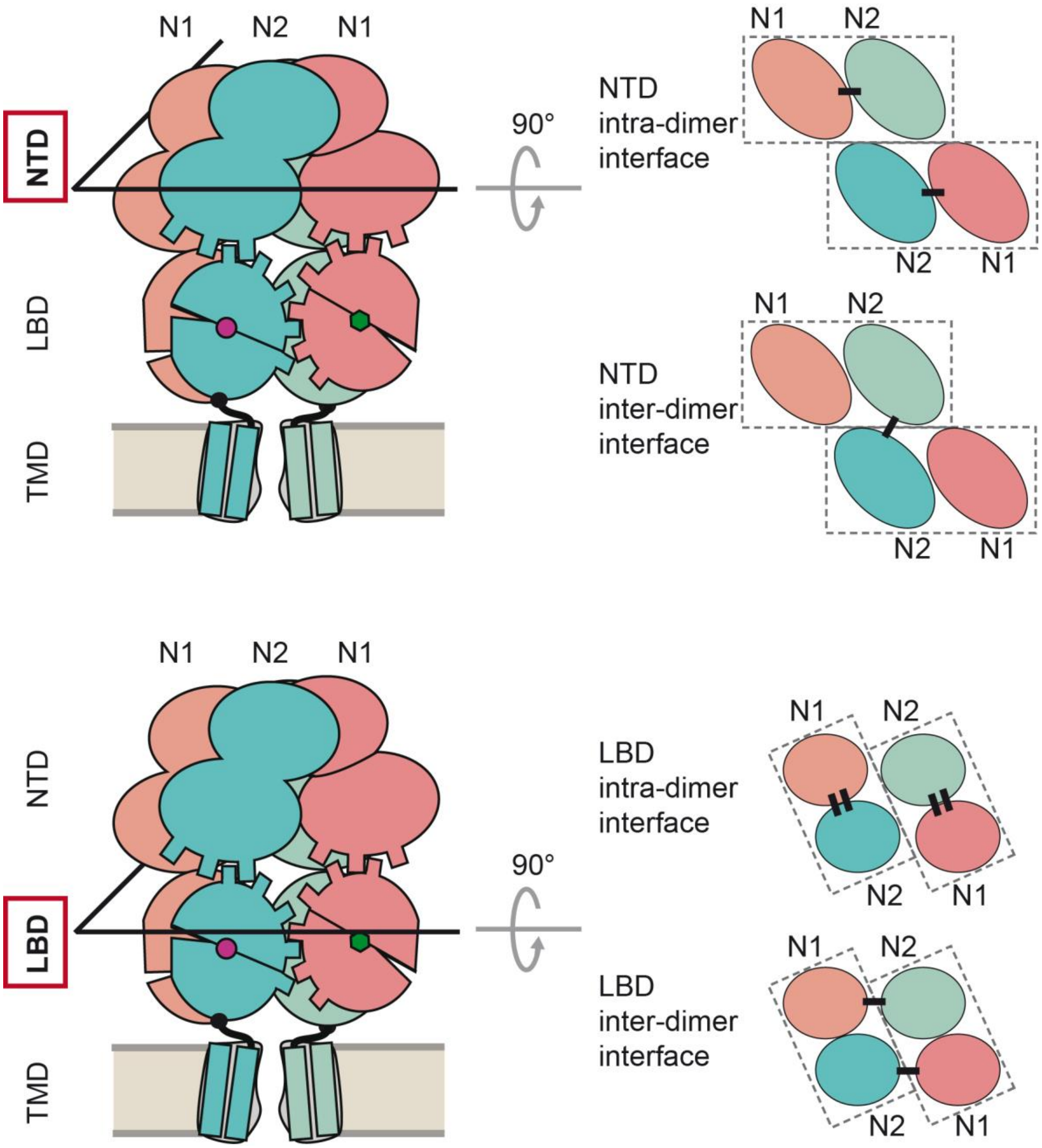

Supplementary Figure 6: Schematic illustration showing the positions for disulfide locking of NTD (upper panel) and LBD (bottom panel) dimers.

Left: side views of the entire receptor. Right: top views of domain organization in the plane highlighted on the left. Dark lines present disulfide bridges (either intra-dimer or inter-dimer). Dashed square shows local dimers in different layers of the receptor.

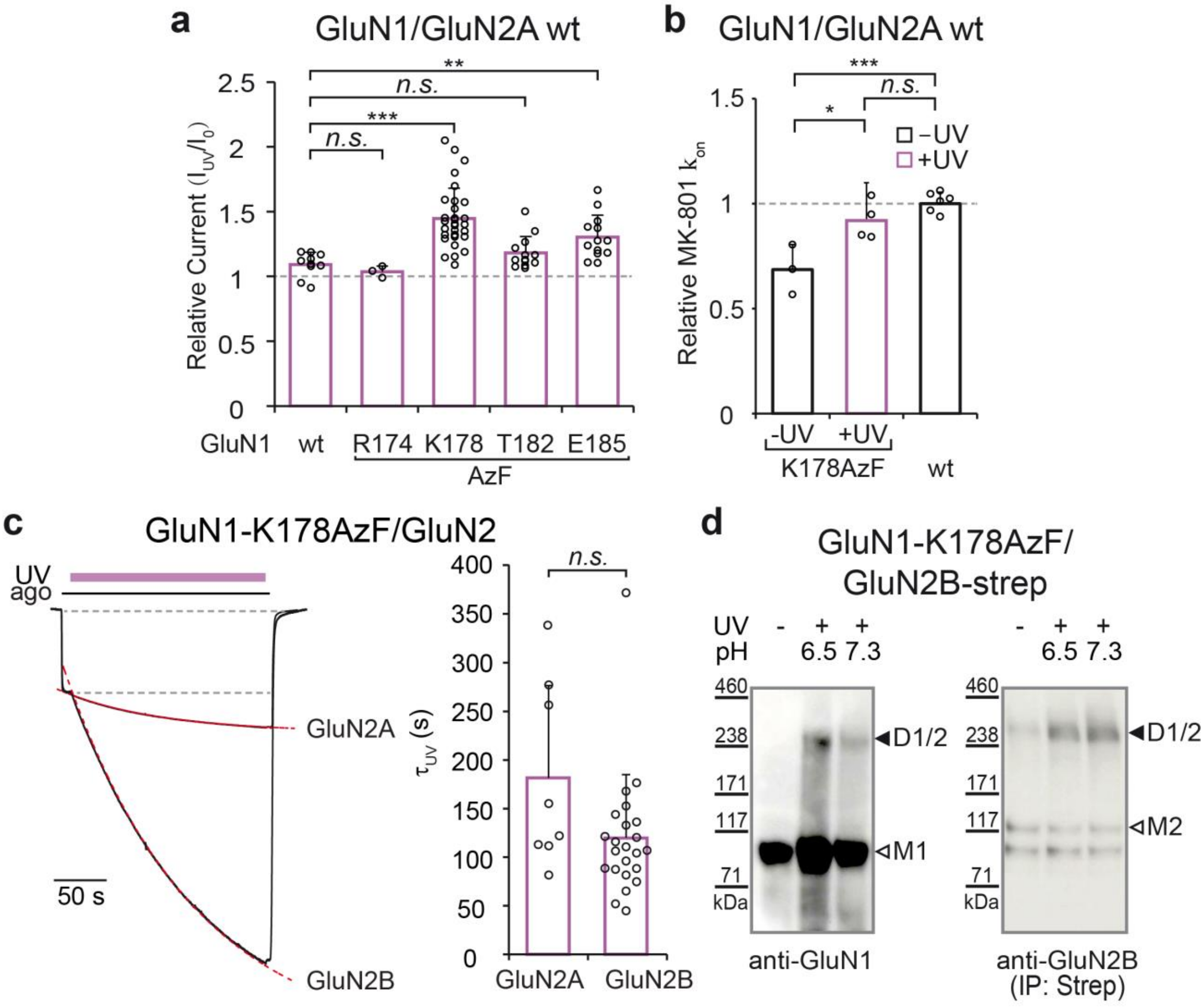

Supplementary Figure 7: Photo-modulation of various GluN1-AzF/GluN2A mutant receptors, time-course of photopotentiation, and photocrosslinking of GluN1-K178AzF/GluN2B receptors at different pH.

(a) Change in current amplitude upon UV illumination (I_{uv}/I_o) of wild-type (wt) GluN1/GluN2A receptors and various GluN1-AzF/GluN2A mutant receptors. Values are: 1.09 ± 0.10 (n=10) for wt; 1.04 ± 0.04 (n=3) for GluN1-R174AzF/GluN2A; 1.45 ± 0.23 (n=32) for GluN1-K178AzF/GluN2A; 1.18 ± 0.13 (n=13) for GluN1-T182AzF/GluN2A; and 1.30 ± 0.17 (n=13) for GluN1-E185AzF/GluN2A. Data represent mean ± SD. n=number of biologically independent cells. **P=0.002, ***P<0.001, *n.s.* non-significant (P=0.36 for GluN1-R174AzF, P=0.07 for GluN1-E185AzF) (one-way ANOVA).

(b) Assessment of receptor channel activity using MK-801 inhibition kinetics. MK-801 k_{on} values were normalized to the mean value obtained with wild-type (wt) GluN1/GluN2A receptors. Relative values are, from left to right: 0.68 ± 0.12, (n=3) without UV and 0.91 ± 0.18 (n=4) with UV for GluN1-K178AzF/GluN2A; 1.00 ± 0.05 (n=6) for wt (no UV). Data represent mean ± SD.

n=number of biologically independent cells. *P=0.032, ***P<0.001 non-significant (P=0.08 for GluN1-K178AzF/GluN2A with UV comparing with wt) (one-way ANOVA).

(c) Kinetics analysis of photo-potentiation. Left: normalized current traces from GluN1-K178AzF/GluN2A and GluN1-K178AzF/GluN2B receptors during UV illumination (365 nm). Dashed red lines show mono-exponential fits. Right: values of mono-exponential fit time constants (τ_{UV}): 182 ± 95 (n=8) for GluN2A receptors *vs* 119 ± 65 (n=23) for GluN2B receptors. Data represent mean ± SD. n=number of biologically independent cells. *n.s*. non-significant (P=0.12) (two-sided Student's t-test).

(d) Immunoblots from HEK cells expressing GluN1-K178AzF/GluN2B receptors. Cells were exposed to UV (+) or not (-) at pH 6.5 or 7.3. Samples were analyzed using anti-GluN1 and anti-Strep antibodies. GluN1 monomer (M1) is expected to run at ~110 kDa, GluN2B-Strep monomer (M2) at ~130 kDa, and GluN1/GluN2B heterodimer (D1/2) ~240 kDa. ‘IP: Strep’ indicates the treatment with an immuno-purification procedure using the Strep tag.

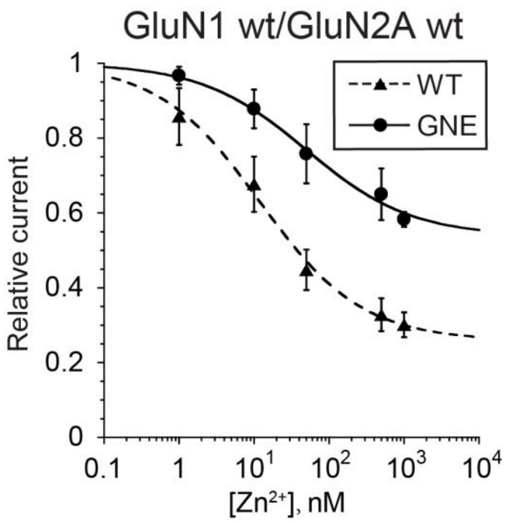

Supplementary Figure 8: The GluN2A PAM GNE-3419 decreased zinc inhibition of GluN1/GluN2A receptors.

Zinc inhibition dose-response curves of wild-type (wt) GluN1/GluN2A receptors in the presence of 100 µM GNE-3419 (GNE, plain line). For comparison, zinc sensitivity of wt GluN1/GluN2A receptors (wt, dashed line) in the absence of GNE is also shown. Values of Zn^{2+} IC_{50}, maximal inhibition and Hill slope (n_H) are: 49.53 ± 33.74 nM, 0.46 ± 0.07 and 0.62 ± 0.17 (n=4-7) in GNE; 12.01 ± 3.36 nM, 0.74 ± 0.04 and 0.64 ± 0.10 (n=8-12) without GNE. Data represent mean ± SD. n=number of biologically independent cells.

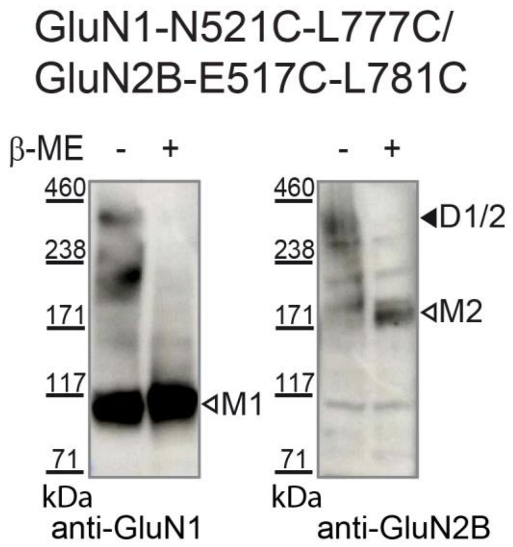

Supplementary Figure 9: Inter-subunit disulfide crosslinking of GluN1-N521C-L777C/GluN2B-E517C-L781C receptors.

Immunoblots from *Xenopus* oocytes expressing GluN1-N521C-L777C/GluN2B-E517C-L781C receptors. Samples were analyzed using anti-GluN1 and anti-GluN2B antibodies. GluN1 monomer (M1) runs at ~110 kDa (M1), GluN2B monomer at ~180 kDa (M2), and GluN1/GluN2B heterodimer at ~290 kDa (D1/2). '± β-ME' indicates immunoblots performed without or with β-mercaptoethanol, i.e. in non- reducing or reducing conditions.

Supplementary references

1. Karakas, E. & Furukawa, H. Crystal structure of a heterotetrameric NMDA receptor ion channel. *Science* 344, 992-997 (2014).
2. Lee, C.-H. et al. NMDA receptor structures reveal subunit arrangement and pore architecture. *Nature* 511, 191-+ (2014).
3. Zhu, S. et al. Mechanism of NMDA receptor inhibition and activation. *Cell* 165, 704-714 (2016).
4. Tajima, N. et al. Activation of NMDA receptors and the mechanism of inhibition by ifenprodil. *Nature* 534, 63-68 (2016).
5. Regan, M.C. et al. Structural Mechanism of Functional Modulation by Gene Splicing in NMDA Receptors. *Neuron* 98, 521-529 e3 (2018).
6. Chou, T.-H., Tajima, N., Romero-Hernandez, A. & Furukawa, H. Structural basis of functional transitions in mammalian NMDA receptors. *Cell* 182, 357-371. e13 (2020).
7. Jalali-Yazdi, F., Chowdhury, S., Yoshioka, C. & Gouaux, E. Mechanisms for zinc and proton inhibition of the GluN1/GluN2A NMDA receptor. *Cell* 175, 1520-1532. e15 (2018).
8. Zhang, J.-B. et al. Structural basis of the proton sensitivity of human GluN1-GluN2A NMDA receptors. *Cell Reports* 25, 3582-3590. e4 (2018).
9. Reddington, S.C. et al. Different photochemical events of a genetically encoded phenyl azide define and modulate GFP fluorescence. *Angewandte Chemie International Edition* 52, 5974-5977 (2013).
