# Supplementary material for: GluN2A and GluN2B NMDA receptors use distinct allosteric routes
Source: Nat Commun. 2021 Aug 5;12:4709. doi: 10.1038/s41467-021-25058-9 (PMC8342458; doi:10.1038/s41467-021-25058-9)
Supplement: Supplementary file 3 — Description of Additional Supplementary Files [file 41467_2021_25058_MOESM3_ESM.pdf]

Description of Additional Supplementary Files

File Name: Supplementary Movie 1
Description: **iMODfit simulations showing the LBD intra-dimer rupture in the GluN1/GluN2A receptor when transiting to the inhibited state.**
At the forefront, the GluN2A subunit is shown in blue with its glutamate-binding LBD in cyan, and the GluN1 subunit is shown in red with its glycine-binding LBD in orange. The movie starts by highlighting the two subunits at positions A (GluN1) and D (GluN2A) within the tetrameric complex. Then the movie shows the modeled trajectory presented in Figure 7 from steps I to III. This trajectory corresponds to a representation of the 'GluN2A route' when the receptor transits from the pre-active to the inhibited state (for further details, see Main Text and Fig. 8). The movie ends by recapitulating step III back and forth.
