# Supplementary material for: GluN2A and GluN2B NMDA receptors use distinct allosteric routes
Source: Nat Commun. 2021 Aug 5;12:4709. doi: 10.1038/s41467-021-25058-9 (PMC8342458; doi:10.1038/s41467-021-25058-9)
Supplement: Supplementary file 5 — Reporting Summary [file 41467_2021_25058_MOESM5_ESM.pdf]

nature research

Corresponding author(s): Pierre PAOLETTI

Last updated by author(s): Jul 19, 2021

Reporting Summary

Nature Research wishes to improve the reproducibility of the work that we publish. This form provides structure for consistency and transparency in reporting. For further information on Nature Research policies, see our Editorial Policies and the Editorial Policy Checklist.

Statistics

For all statistical analyses, confirm that the following items are present in the figure legend, table legend, main text, or Methods section.

n/a	Confirmed	
☐	☒	The exact sample size (*n*) for each experimental group/condition, given as a discrete number and unit of measurement
☐	☒	A statement on whether measurements were taken from distinct samples or whether the same sample was measured repeatedly
☐	☒	The statistical test(s) used AND whether they are one- or two-sided<br>*Only common tests should be described solely by name; describe more complex techniques in the Methods section.*
☒	☐	A description of all covariates tested
☒	☐	A description of any assumptions or corrections, such as tests of normality and adjustment for multiple comparisons
☐	☒	A full description of the statistical parameters including central tendency (e.g. means) or other basic estimates (e.g. regression coefficient) AND variation (e.g. standard deviation) or associated estimates of uncertainty (e.g. confidence intervals)
☐	☒	For null hypothesis testing, the test statistic (e.g. F, t, r) with confidence intervals, effect sizes, degrees of freedom and P value noted<br>*Give P values as exact values whenever suitable.*
☒	☐	For Bayesian analysis, information on the choice of priors and Markov chain Monte Carlo settings
☒	☐	For hierarchical and complex designs, identification of the appropriate level for tests and full reporting of outcomes
☒	☐	Estimates of effect sizes (e.g. Cohen's *d*, Pearson's *r*), indicating how they were calculated

Our web collection on statistics for biologists contains articles on many of the points above.

Software and code

Policy information about availability of computer code

Data collection	Clampex (10.5 & 10.6)
Data analysis	Kaleidagraph 4.0, PyMol (0.99), Clampfit (10.5)

For manuscripts utilizing custom algorithms or software that are central to the research but not yet described in published literature, software must be made available to editors and reviewers. We strongly encourage code deposition in a community repository (e.g. GitHub). See the Nature Research guidelines for submitting code & software for further information.

Data

Policy information about availability of data

All manuscripts must include a data availability statement. This statement should provide the following information, where applicable:

- Accession codes, unique identifiers, or web links for publicly available datasets
- A list of figures that have associated raw data
- A description of any restrictions on data availability

- PDB 4PE5; https://www.rcsb.org/structure/4PE5
- PDB 6MMR; https://www.rcsb.org/structure/6MMR
- All web-links to publicly available PBD codes are inserted in the manuscript.
- All data are openly accessible in the provided source data file.

April 2020

Field-specific reporting

Please select the one below that is the best fit for your research. If you are not sure, read the appropriate sections before making your selection.

- [x] Life sciences
- [ ] Behavioural & social sciences
- [ ] Ecological, evolutionary & environmental sciences

For a reference copy of the document with all sections, see nature.com/documents/nr-reporting-summary-flat.pdf

Life sciences study design

All studies must disclose on these points even when the disclosure is negative.

Sample size	No statistical methods were used to pre-determine sample size. Electrophysiology and immunoblots sample sizes were chosen based on previous experience with data of this sort, and reproducibility of results across independent experiments. The authors have extensive previous experience with data of this type (e.g. Zhu et al., PNAS 2014; Grand et al., Klippenstein et al., eLife 2017; Nat Comm 2018; Esmenjaud et al., EMBOJ 2019). Hence, sample size were based on understanding of sample variabilities in the field (for electrophysiology, usually n numbers between 3 and a few tens; for immunoblots, at least two independent repeats).
Data exclusions	No data were excluded from the study
Replication	All experiments were repeated several times with different batches of cells and chemicals to ensure reproducibility. Number of repeats are systematically included in the manuscript as n numbers in the figure legends (with definition of n also included). All immunoblots were repeated in independent experiments at least twice, as stated in the Methods section. All attempts at replication were successful.
Randomization	Randomization is not relevant to electrophysiology or immublotting experiments as samples were not divided into experimental groups. Mutant receptors were systematically compared/processed in parallel to control wild-type (WT) receptors. Moreover, data were systematically double-checked by another observer and contributor of the study.
Blinding	No blinding was performed but mutant receptors were systematically compared/processed in parallel to control wild-type (WT) receptors. Moreover, data of experiments were systematically double-checked by another observer and contributor of the study.

Reporting for specific materials, systems and methods

We require information from authors about some types of materials, experimental systems and methods used in many studies. Here, indicate whether each material, system or method listed is relevant to your study. If you are not sure if a list item applies to your research, read the appropriate section before selecting a response.

Materials & experimental systems

n/a	Involved in the study	
[ ]	[x]	Antibodies
[ ]	[x]	Eukaryotic cell lines
[x]	[ ]	Palaeontology and archaeology
[x]	[ ]	Animals and other organisms
[x]	[ ]	Human research participants
[x]	[ ]	Clinical data
[x]	[ ]	Dual use research of concern

Methods

n/a	Involved in the study	
[x]	[ ]	ChIP-seq
[x]	[ ]	Flow cytometry
[x]	[ ]	MRI-based neuroimaging

Antibodies

Antibodies used	anti-GluN1 (Millipore, mouse monoclonal MAB1586, clone R1JHL, Lot:3050817,3482889,3352449)<br>anti-GluN2A (Millipore, rabbit monoclonal 04-901, clone A12W, Lot: 2949958,3239893)<br>anti-GluN2B (NeuroMab, mouse monoclonal 75-101 clone N59/36, Lot: 455-10JD-82)<br>anti-Strep (Abcam, rabbit monoclonal ab180957 clone EPR12666, Lot: GR3212622-2)<br>Peroxidase-conjugated anti-mouse antibody (Jackson ImmunoResearch, goat polyclonal, #115-035-003)
Validation	anti-GluN1 (specificity to all splice variants of GluN1; validated for use in WB; species reactivity: human, mouse and rat; used in 5 papers according to manufacturer's website)<br>anti-GluN2A (specificity to GluN2A; suitable for WB, species reactivity: rat and mouse; used in 22 papers according to manufacturer's website)<br>anti-GluN2B (does not cross-react with GluN2A, application: IHC, IP, WB; species reactivity: human, mouse and rat; validated in WB, immuno-cytochemistry and immuno-histochemistry and has been knockout-validated in mouse brain according to manufacturer's website)<br>anti-Strep (suitable for WB; species independent; has been referenced in 1 paper according to manufacturer's website)

Eukaryotic cell lines

Policy information about cell lines

Cell line source(s)	HEK-293 cells (human kidney cell line, catalogue no. 96121229 from European Collection of Authenticated Cell Cultures (ECACC).
Authentication	The cell line was not authenticated
Mycoplasma contamination	The cells have been eradicated from mycoplasma at ECACC.
Commonly misidentified lines (See ICLAC register)	No commony misidentified cell lines were used in this study
